# Hypotension artérielle intra dialytique chez un hémodialysé chronique révélatrice d'insuffisance antéhypophysaires

**DOI:** 10.11604/pamj.2016.24.50.8813

**Published:** 2016-05-11

**Authors:** Jaouad El Maghraoui, Hanane Ouahabi, Nadia Kabbali, Mohamed Arrayhani, Farida Ajdi, Tariq Sqalli Houssaini

**Affiliations:** 1 Service de Néphrologie, CHU Hassan II, Equipe de Recherche REIN, Faculté de Médecine et de Pharmacie, Fès, Maroc; 2 Service d'Endocrinologie et Maladies Métaboliques, CHU Hassan II, Fès, Maroc

**Keywords:** Hémodialysé chronique, hypotension per dialytique, insuffisance antéhypophysaire, craniopharyngiome

## Abstract

L'hypotension artérielle per dialytique est une complication fréquente chez l'hémodialysé chronique. Elle est occasionnée par des facteurs liés à la séance d'hémodialyse et/ou au patient. Nous rapportons le cas d'un patient âgé de 42 ans, hémodialysé chronique sur néphropathie lithiasique depuis 5 ans. Il a rapporté des céphalées chroniques atypiques, compliquées d'une baisse progressive de l'acuité visuelle, une asthénie, une hypertrophie mammaire, et une baisse de libido. Il est référé pour une hypotension artérielle per dialytique non expliquée par les causes habituelles, dont la recherche étiologique a objectivé une insuffisance anté hypophysaire et une masse hypophysaire à l'IRM. A travers cette observation, nous montrons qu'après avoir éliminé les causes classiques d'hypotension artérielle chez l'hémodialysé, une cause endocrinienne doit être recherchée.

## Introduction

L'hypotension artérielle per dialytique est une complication fréquente chez l'hémodialysé chronique. Elle se définit par une pression artérielle systolique < 100 mm Hg ou une chute de la pression artérielle diastolique supérieure à 20 mm Hg avec des symptômes associés. Elle est occasionnée par des facteurs liés à la séance d'hémodialyse et/ou au patient. Nous rapportons à travers ce cas l'intérêt de la recherche d'une cause endocrinienne devant l'hypotension artérielle chez l'hémodialysé chronique non expliquée par les causes habituelles.

## Patient et observation

**Figure 1:**
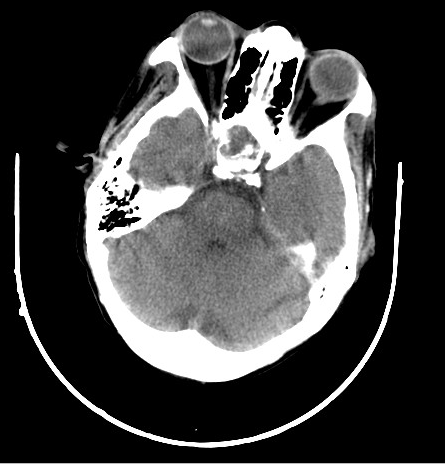
TDM cérébrale objectivant une macro adénome hypophysaire agressif

**Figure 2:**
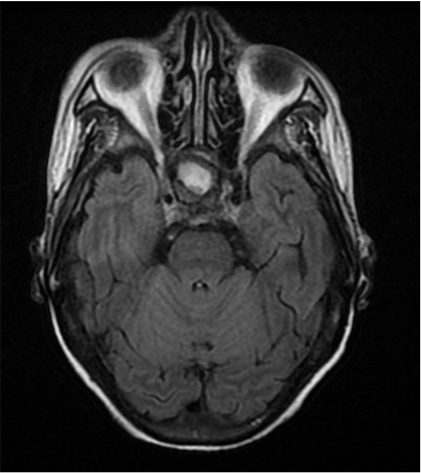
Coupe axiale de l'IRM cérébrale objectivant une masse hypophysaire

**Figure 3:**
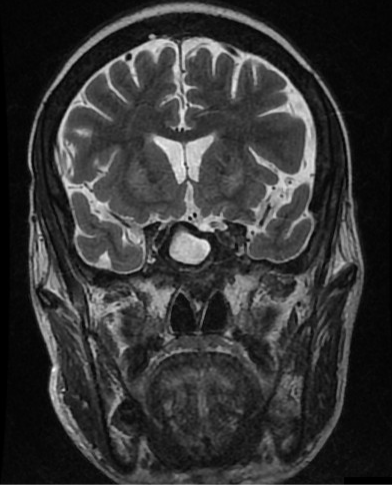
Coupe coronale de l'IRM cérébrale objectivant une masse hypophysaire

**Figure 4:**
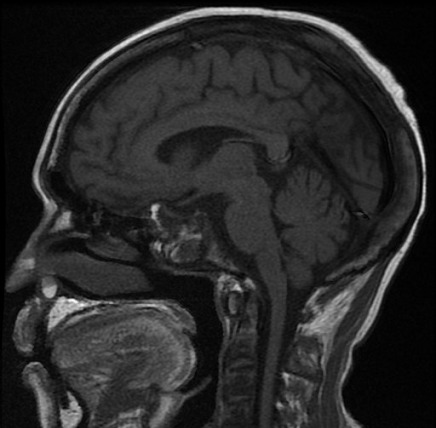
Coupe sagittale de l'IRM cérébrale objectivant une masse hypophysaire

Il s'agit d'un patient, âgé de 45 ans, hémodialysé chronique sur néphropathie lithiasique depuis cinq ans rapportant une notion de céphalées chroniques atypiques, compliquées d'une baisse progressive de l'acuité visuelle, une asthénie, une hypertrophie mammaire bilatérale et une baisse de libido, référé pour une hypotension artérielle per dialytique non expliquée par les causes habituelles. L'examen clinique trouve une hypotension artérielle à 60/50 mm Hg, une bradycardie à 54bpm avec un examen cardiovasculaire normal et une hypoglycémie à 0.6g/dl. Il s'agit d'un patient imberbe, ayant une gynécomastie bilatérale stade II sans écoulement lactescent ou sanglant. L'examen des caractères sexuels secondaires a objectivé une verge de 8 cm, des testicules intra scrotaux bilatéral chacun est de 4/3 cm, avec une pilosité pubienne stade III. L'examen cervical était normal, avec notamment absence de goitre, de nodules thyroïdiens, et d'adénopathies cervicales. Ce tableau clinique a justifié la réalisation d'un bilan hormonal à la recherche d'une pathologie endocrinienne. Ainsi nous avons noté une insuffisance corticotrope, gonadotrope, somatotrope associé à une hyperprolactinémie; évoquant un adénome à prolactine vu le taux tumoral de la prolactinémie, ou une hyperprolactinémie de déconnection aggravée par l'insuffisance rénale (Tableau 1). Par ailleurs, le patient a bénéficié d'une TDM cérébral ayant objectivé une macro adénome hypophysaire agressif mesurant 36x25x32 mm (Figure 1). La TDM cérébrale a été complété par une IRM hypophysaire qui a mis en évidence une masse tumorale sellaire et supra sellaire à triple composante liquidienne, calcique et charnue évocatrice d'un craniopharyngiome (Figure 2, Figure 3, Figure 4). L’évolution a été marquée par l'amélioration clinique et biologique du patient avec notamment la normalisation de la pression artérielle et de la fréquence cardiaque et correction du déficit corticotrope et thyréotrope (Tableau 2).

**Tableau 1:** Exploration biologique des différentes axes hypophysaires

Axe	Hormone	Taux
Corticotrope	Cortisol de 8h	51.45µg / l [100-250]
Thyréotrope	TSH	6.1µU /L [0,25-3,8]
	LT3	1pg/ml [0,7-1,6]
	LT4	40pmol/l [45-120]
	AC anti TPO	0.25U/ml [N < 60U/ml]
Gonadotrope	FSH	<0.05U/L [1,4-18,1]
	LH	0.01U/L [1,5-9,3]
	Testostérone totale	0.99µU/ml [2,8-11]
Prolactinémique	Prolactine	>200ng/ml [2-15]
Somatotrope	GH	0.04ng/ml [1-4]

**Tableau 2:** Évaluation clinique et biologique du patient après correction des déficits hormonaux

Evaluation clinique et biologique	Avant	Après un mois
Tension artérielle	60/50 mmHg	100/60 mmHg
Fréquence cardiaque	54 bpm	72 bpm
Glycémie	0,51g/l	1,34 g/l
TSH	10,6 mui/l	3,4 mui/l
Cortisol de 8h	51.45 µg /l	132 µg /l

## Discussion

De nombreux facteurs sont impliqués dans la survenue des épisodes d'hypotensions dialytiques: le patient et sa comorbidité, les traitements en cours, les conditions de dialyse et surtout la perte de poids horaire [1, 2]. Le mécanisme commun qui sous-tend l'hypotension dialytique est la rupture de l’équilibre entre le débit cardiaque, qui tend à diminuer du fait de la réduction du volume plasmatique, et les résistances vasculaires périphériques, lorsqu'elles ne peuvent s’élever assez rapidement pour compenser la baisse du débit cardiaque [3, 4]. Le pan hypopituitarisme se définit par le déficit de la plupart des hormones de l'hypophyse antérieur y compris LH, FSH, ACTH, TSH et GH. Ainsi le pan hypopituitarisme entraine une hypothyroïdie, fonction corticosurrénalienne réduite et hypogonadisme et être accompagnée par la perte de poids, perte de l'appétit, faiblesse, hypotension artérielle, la fatigue, perte de mémoire, et la dépression [5, 6]. Le diagnostic différentiel d'une masse hypophysaire comprend un cancer métastatique, des hémopathies malignes, granulomatoses et infections [7]. Les tumeurs malignes métastatiques et les lymphomes atteignent surtout l'hypophyse postérieure et se manifestent le plus souvent par le diabète insipide. Les adénomes hypophysaires touchent l'hypophyse antérieure et se manifestent par hypopituitarisme [8, 9]. Dans notre cas, l'hypotension artérielle a été secondaire à un déficit corticotrope [10] suite à une masse tumorale hypophysaire qui a comme conséquences une insuffisance antéhypophysaire associé à une hyperprolactinémie [11]. Après la correction des déficits hormonaux le patient était amélioré sur le plan clinique et biologique.

## Conclusion

Devant toutes ces manifestations cliniques et biologiques le diagnostic d'une insuffisance antéhypophysaire avec hyperprolactinémie a été retenu. A travers cette observation, nous montrons qu'après avoir éliminé les causes classiques d'hypotension artérielle chez l'hémodialysé, une cause endocrinienne doit être recherchée.
